# Parametric delineation uncertainties contouring (PDUC) modeling on CT scans of prostate cancer patients

**DOI:** 10.1002/acm2.13970

**Published:** 2023-04-20

**Authors:** Vi Ly, Lizhong Liu, Carlos Cardenas, Sean Maroongroge, Brian De, Daniel El Basha, Laurence Court, Xi Luo

**Affiliations:** ^1^ Division of Radiation Oncology The University of Texas MD Anderson Cancer Center Houston Texas USA; ^2^ Department of Biostatistics and Data Science University of Texas Health Science Center School of Public Health Houston Texas USA

**Keywords:** cancer, contouring, delineation uncertainties, simulation algorithm, treatment planning

## Abstract

**Purpose:**

Variability in contouring contributes to large variations in radiation therapy planning and treatment outcomes. The development and testing of tools to automatically detect contouring errors require a source of contours that includes well‐understood and realistic errors. The purpose of this work was to develop a simulation algorithm that intentionally injects errors of varying magnitudes into clinically accepted contours and produces realistic contours with different levels of variability.

**Methods:**

We used a dataset of CT scans from 14 prostate cancer patients with clinician‐drawn contours of the regions of interest (ROI) of the prostate, bladder, and rectum. Using our newly developed Parametric Delineation Uncertainties Contouring (PDUC) model, we automatically generated alternative, realistic contours. The PDUC model consists of the contrast‐based DU generator and a 3D smoothing layer. The DU generator transforms contours (deformation, contraction, and/or expansion) as a function of image contrast. The generated contours undergo 3D smoothing to obtain a realistic look. After model building, the first batch of auto‐generated contours was reviewed. Editing feedback from the reviews was then used in a filtering model for the auto‐selection of clinically acceptable (minor‐editing) DU contours.

**Results:**

Overall, C values of 5 and 50 consistently produced high proportions of minor‐editing contours across all ROI compared to the other C values (0.936 ±0.111 and 0.552 ±0.228, respectively). The model performed best on the bladder, which had the highest proportion of minor‐editing contours (0.606) of the three ROI. In addition, the classification AUC for the filtering model across all three ROI is 0.724 ±0.109.

**Discussion:**

The proposed methodology and subsequent results are promising and could have a great impact on treatment planning by generating mathematically simulated alternative structures that are clinically relevant and realistic enough (i.e., similar to clinician‐drawn contours) to be used in quality control of radiation therapy.

## 1 INTRODUCTION

In the medical imaging field, segmentation is a popular image processing for the task of segregating the target regions of interest (ROI) from the background of the image.^1^ Research in image segmentation has resolved technical challenges such as fuzzy image elucidation or few‐show learning with limited data.^2^, ^3^, ^4^ In this paper, we are particularly interested in a related but different problem of automatically generating a multitude of random contours with varying clinical relevance, such as clinical usability in treatment and resemblance to clinicians’ hand‐drawn contours. Indeed, contouring is a crucial step in radiation treatment planning, as any errors will lead to treatment errors (i.e., systematic errors) and inaccurate calculations of dose‐volume histograms (DVHs) and tumor‐control probability (TCP). However, one particular issue that is often overlooked by researchers when it comes to contouring is uncertainties.^5^

Contouring has several inherent uncertainties owing to factors such as the microscopic spread of disease during treatment, patient set‐up error, organ movement, and patient movement. However, the leading factor in geometric uncertainties is still inter‐observer delineation variability.^6^ Many studies have found significant inter‐observer variability in contouring for all anatomies and treatment sites.^7^, ^8^ In particular, inter‐observer contouring variability has a statistically significant impact on the dosimetric parameters in the lower abdominal region (prostate, bladder, rectum).^9^ Although there is reasonable agreement among clinicians about the contouring of the prostate,^10^, ^11^ Seddon et al.^10^ found a great deal of variation in the outlining of the prostatic apex and the rectum among the clinicians. In addition, Lebesque et al.^12^ reported large differences in the contours of the bladder drawn by different observers.

Bernstein et al.^13^ [Correction added on May 1, 2023, after first online publication: Reference citation was corrected from David et al.] found that the process of manually flagging incorrect or suspicious contouring was highly labor‐intensive and time‐consuming. It is also a challenging task for clinicians to draw DU contours without introducing bias. Because of this, several groups are working to develop tools to automatically flag incorrect contouring^14^ and to minimize these errors.^10^, ^11^, ^12^, ^13^, ^14^, ^15^, ^16^, ^17^ This task can be automatically achieved in a shorter time without any biases using mathematical modeling. However, DUs are not easily modeled. Owing to a limited number of manual contours and a tedious manual validation process, most DU research has explored DU through 3D visualization analysis.^10^, ^11^ Some researchers have also explored mathematical simulation modelings such as the Monte Carlo method,^15^, ^16^, ^17^ stochastic programming,^20^ and worst‐case robust optimization.^21^ Xu et al.^22^ have introduced the Average Surface of Standard Deviation (ASSD) model, which estimates the voxel‐specific displacement vector to determine the alternative ROI structures based on the physician‐contoured volume. This model produced qualified delineated errors for coverage probability evaluations.

A significant hurdle when developing tools to identify poor contours is the lack of good datasets (with labeled contours of various qualities). To address this, we propose utilizing the concept of delineated‐uncertainties (DU) contouring. This paper defines DU contouring as outlining the uncertainties around the boundaries between gross tumors and normal tissue in medical imaging. The delineation of these ROI, which are the interface between target volumes and surrounding organs at risk (OAR), is critically important for quality control. Intentional generation of DU contours can be useful for the task of building a large dataset of alternative ROI. A large dataset of various organ structures from the same computed tomography (CT) images improves the quality of image‐guided radiation therapy (IGRT) in terms of target localization.

Because our focus is on DU contouring of the ROI, we found that the ASSD model is more relevant to our project than other mathematical methods such as stochastic and Monte Carlo models that incorporate dosage information in their formulations. However, a common criticism of the ASSD is that its output is in the form of displacement vectors rather than an actual geometric contour. This is due to the fact that it was built for the sole purpose of coverage probability evaluations.^22^

To overcome the limitations of the ASSD model, we proposed a novel method called Parametric Delineation Uncertainties Contouring (PDUC). By mimicking the phenomenon of the interobservers’ geometric uncertainties, our approach can automatically generate several alternate DU contours and save a great amount of time if the task was performed by human labor. Two main novel contributions in our method are: Firstly, since the model is parametric, we can control the level of variations on the generated contours; Secondly, the generated DU contours are realistic and smooth as if they are drawn by a clinician. In our paper, we discuss the details of the PDUC model‐building process and its components. In addition, we also assess the model performance in various parameter settings. To the best of our knowledge, this study would serve as the first exploration and evaluation of any parametric DU contouring models for organs within the lower abdominal area.

## 2 METHODS

**FIGURE 1:**
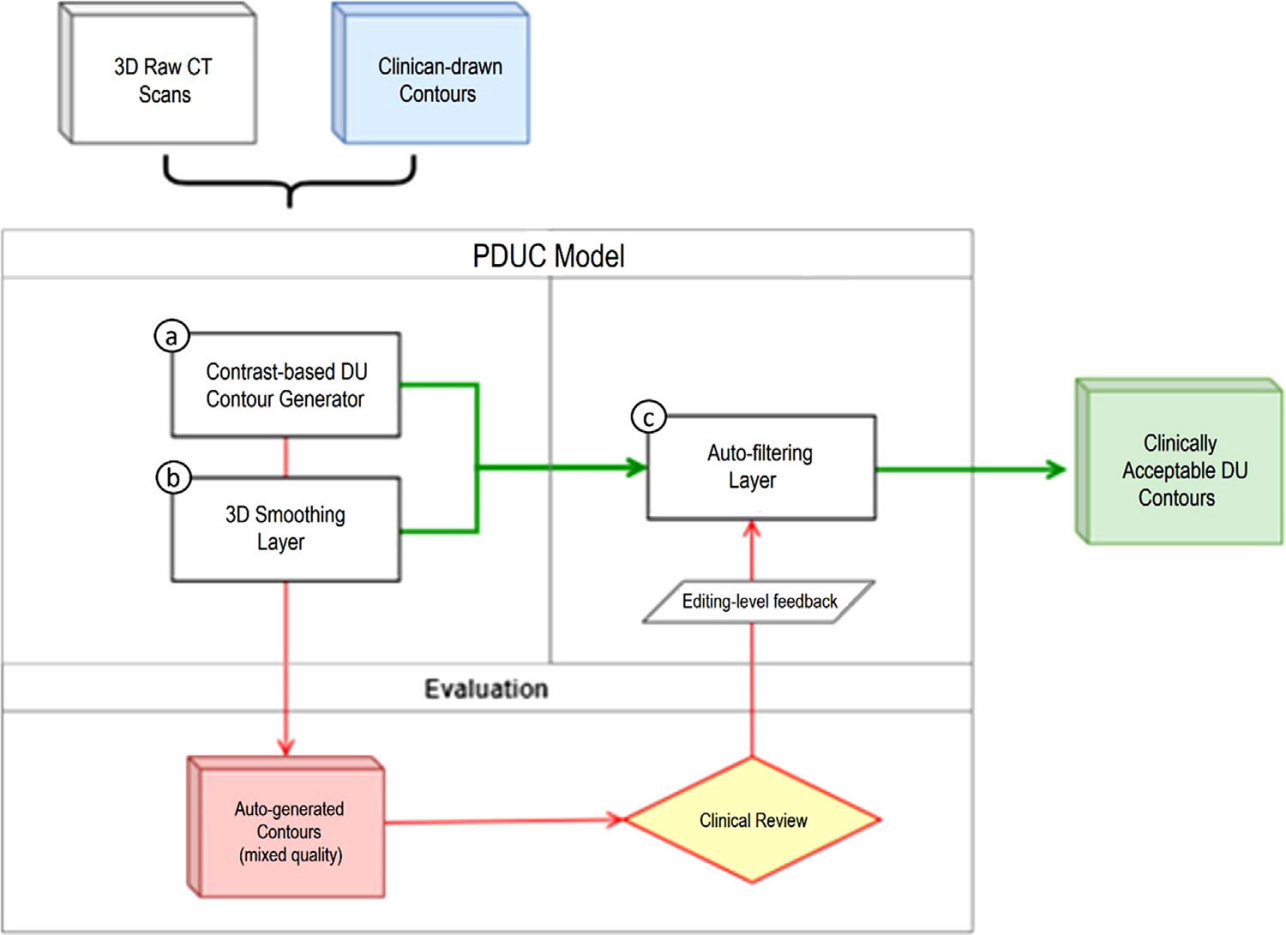
PDUC model building and evaluation process. The raw images and clinician hand‐drawn contours were passed through an (a) contrast‐based DU generator and (b) a 3D smoothing layer. Then, the generated batch of DU contours of mixed quality was sent for clinical review. The feedback from the review was used to develop the (c) auto‐filtering layer. The auto‐filtering layer helps filtering out unacceptable contours and selecting only acceptable contours for clinical use. The outputs are binary masks representing the DU contours.

To develop our model, we used a dataset of CT scans from 14 prostate cancer patients. In our study, we include subjects who have all 3 ROI of the prostate, bladder, and rectum presented in the CT scans. Thus, we exclude subjects who are labeled “prostate‐fossa,” which means the subject have their prostate removed due to cancer. In addition, the prostate was considered the primary target volume while the bladder and rectum were the surrounding OARs. In our model‐building process (red path in Figure 1), CT scans with clinician‐drawn (clinician hand‐drawn) contours were fed through the PDUC model, which consisted of a contrast‐based DU generator (Section 2A) and a 3D smoothing layer (Section 2B), as described in Figure 1. The first batch of auto‐generated contours was sent to clinical reviewers to assess the “editing level” that would be required for clinical use. This feedback was later used as the target outcome in building a filtering model for the auto‐selection of clinically acceptable (some minor edits required) DU contours. This filtering model was eventually integrated into the final main pipeline (green path in Figure 1) for a seamless process of auto‐generating and auto‐selection of acceptable DU contours. The outputs are binary masks representing the DU contours. This pipeline is our final version of the PDUC model for application in the clinical setting.

A. Contrast‐based DU generator:

DU contours were generated by deforming, contracting, and expanding the ground‐truth contours. The PDUC model uses image contrast as the source of contouring delineation variations. Hence, all the transformations (deformation, contraction, or expansion) are written as functions of image contrast in voxels on and adjacent to the ground‐truth‐contoured surface. Since our task was contouring, we built the model to account for only the surface voxels. To mimic the clinician's slice‐to‐slice contouring method in real‐life, the model is designed for 2D CT slices rather than 3D images as in the ASSD model. Contouring on 2D slices also allows us to implement cropping on the target region and multithreading to process multiple slices at once. This helps the PDUC algorithm become lighter for future deployment. Hence, it can be easily implemented by a broad audience, including clinicians in different developing countries with various conditions of computing power. Even though these CT slices were two‐dimensional, they had a fixed thickness and so were considered to contain voxels, not pixels. Since the thickness of each voxel was fixed, we only focused on calculating the displacement vector along the *x* and *y* directions of the slice. As a result, the magnitude of the DUs for each surface ROI pixel from the ground‐truth slices was scaled as an alternative location by a voxel‐specific displacement vector, ${\vec{D}}_{i,\;j,}\;$by applying some random base contouring uncertainty variables (or variations), $W_{i,\;j}\left(C\sigma \right),$ and truncating Gaussian function, $TG(s,\;s_{0},\;w_{0}),\;$to the voxel‐specific CT image intensity, ${\tilde{F}}_{ct}\left(s\right).$

(1) $${\vec{D}}_{i,\;j};\;=\;\left[W_{i,\;j}\left(C\sigma \right)\cdot TG\left(s,\;s_{0},\;w_{0}\right)\right]\cdot {\tilde{F}}_{ct}\left(s\right)$$

To find ${\tilde{F}}_{ct}\left(s\right)$, we first used the Sobel Operator Gradient function (Grad) with kernel 3 × 3 to compute the CT number gradient of a voxel in the k direction, G(s), for all the relevant voxels on the surface.

(2) G(s)=Grad[L(s)]

Then we calculated the CT image intensity gradient of each voxel, $F_{CT}\left(s\right)$, using Equation (3) with base gradient *a* = 50. If a has a small value like 1, F_CT_(s) will be only sensitive to very low gradient (up to only 20 HU/mm), while a large value of a like 10 000 may not be effective since the F_CT_ *(s)* would not change with different levels of gradients. Therefore, *a* = 50 is a reasonable selection.^22^ Letting *L* be the list of voxel indexes of the ROI surface on a certain slice ROI_z_, and *s* is a voxel index on *L*.

(3) $$F_{CT}\left(s\right)=\frac{a}{|G(s)|+a}$$

The last step to compute ${\tilde{F}}_{ct}\left(s\right)$, we average the CT image intensity gradient of 30 nearby target surface voxels in the *x* and *y* directions as in (4). This averaging helps soften the sharp edges produced by the displacement vector $\vec{D}$ in the *x* and *y* space. Hence, the mathematically obtained contour can approximate the shape of a contoured hand drawn by a human clinician.

(4) $$\begin{array}{ccc} & & {\tilde{F}}_{\mathrm{ct}}\left(s\right)=\frac{1}{2k+1}\\ & & \;\left[F_{\mathrm{CT}}\left(s\right)+\sum_{i=1}^{k}\left(F_{\mathrm{CT}}\left(s+i\right)+F_{\mathrm{CT}}\left(s-i\right)\right)\right]\;,\text{where}\;k=15.\; \end{array}$$

$\mathrm{For}\;\mathrm{variation}\;W_{i,\;j}\left(C\sigma \right)\;\mathrm{of}\;\mathrm{each}\;\mathrm{voxel}$, we computed the variable using an inverse standard normal cumulative density function.

(5) $$W_{i,\;j}\left(C\sigma \right)=norminv\left(p_{i,\;j},\;0,\;C\sigma \right)$$

with random probability $p_{i,\;j}$, mean 0, and standard deviation Cσ.Here, C is a constant that can be adjusted to modify the variation of the DU contour, and σ is the ROI‐specific standard deviation.^22^

Lastly, we need to introduce the $TG(s,\;s_{0},\;w_{0}).\;$The previous functions of ${\tilde{F}}_{ct}\left(s\right)$ and $W_{i,\;j}\left(C\sigma \right)\;$ are calculated on each individual slice, inconsistency of generated contours between slices may arise. Hence, the truncated Gaussian (TG) function helps ensure that the calculation of a voxel displacement vector in a slice incorporates the voxel location information from all other the slices in the ROI as in the following equation:

(6) $$\begin{array}{ccc} & & TG\left(s,\;s_{0},\;w_{0}\right)=\frac{1}{\sqrt{2\pi }w_{0}}\\ & & exp\left(-\frac{{\left[min\left(\left|s\;-\;s_{0}\right|,R-\left|s-s_{0}\right|\right)\right]}^{2}}{2w_{0}^{2}}\right) \end{array}$$

where *s*_0_ is found using the 3D Perturbation Algorithm, *w*_0_ is *R/5*, for any s=1,2,…,R, with *R* being the number of voxels on *L*. The minimal function $min(|s\;-\;s_{0}\left|,R-\right|s-s_{0}|)$ calculates the minimal circular distance between indices *s* and *s*_0_ along *L*.

**Table:** 

3D Perturbation Algorithm
(i) Assume that each ROI consists of n slices, and each slice represents a connected region. Let slices of ROI be ROI_z_, where *z = 0, 1, …, n*;(ii) Pick slice 0 as the starting slice, and calculate the average gradient using the following equations:(a) Let ${\left(x_{z},\;y_{z}\right)}_{r}$ be *r*th voxel on L of slice *z*, find the voxel ${\left(x_{z+1},\;y_{z+1}\right)}_{r}$ on slice z+1 such that $∥x_{z}-x_{z+1}{∥}^{2}+{∥y_{z}-y_{z+1}∥}^{2}$ is the smallest; (b) For each voxel *r*, we repeat the previous step to build a list of voxels as follows: $\Phi \left(r\right)\;=\;\left[{\left(x_{0},\;y_{0}\right)}_{r},\;{\left(x_{1},\;y_{1}\right)}_{r},\;{\left(x_{2},\;y_{2}\right)}_{r},\;\text{…},\;{\left(x_{n},\;y_{n}\right)}_{r}\right]$ (c) For each voxel *r*, calculate the average gradient as $$\bar{\mathrm{avg}\_\mathrm{gra}d_{r}}=\frac{1}{\left|\Phi (r)\;\right|}\sum_{z=0}^{n}\text{APTARAHARPOON}\hat{\mathrm{Grad}}{\left(x_{z},y_{z}\right)}_{r}.$$ Pick voxel *s*_0_ such that $\bar{avg\_grad_{r}}$ at $r\;=s_{0}\;\;$is the smallest in magnitude and use this *s*_0_ in the TG function (6).

**FIGURE 2:**
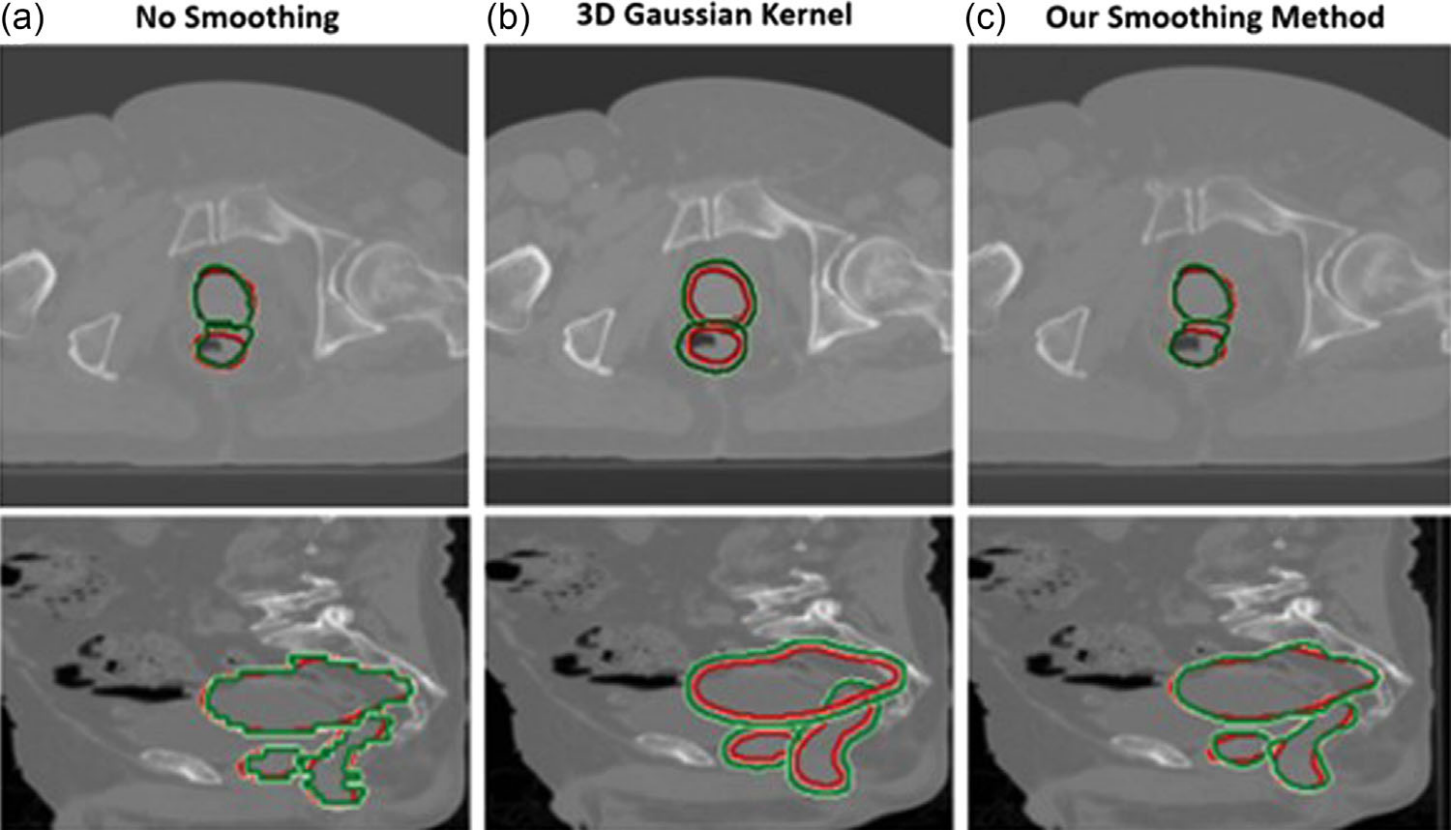
Effect of different smoothing filters on the generated contour. Contours were generated (a) without any smoothing, (b) after applying 3D Gaussian kernel smoothing kernel, and (c) our smoothing method.

B. Smoothing layer:

In this section, we define a smoothing layer using Gaussian kernels which further improve the slice‐to‐slice smoothness of the generated DU contours. We have experimented with using a 3D Gaussian kernel and found that this type of kernel constantly resulted in overly smoothed contours. This over‐smoothing effect is not desirable since it would lead to a loss of distinct variations produced by the contrast‐based DU generator (described in section A). We demonstrated an example of the over‐smoothed contours from the 3D kernel in Figure 2. To overcome this over‐smoothing problem, we apply the filter on the (*x*, *y*) dimension, then on the (*x*, *z*) direction. This smoothing method partly mimics how a clinician would contour in real life. First, he/she may contour the organ slice by slice (axial view), and later correct the contour in the (*x*, *z*) direction as they look at the sagittal view of the organ.

As we generate the contours from slice to slice along the (*x, y*) dimension, the result might not be consistent across the z‐axis of ROI. This would be particularly noticeable to a clinician viewing sagittal or coronal slices. Hence, there is a need for a smoothing algorithm. To smooth out the contours produced by mathematically displaced ROI voxels, we used the method of a 2D Gaussian smoothing filter. Since each 2D slice has an x and y direction. The Gaussian blur function is applied to each slice following this formula:

(7) $$G\left(x,\;y\right)=\frac{1}{2\pi \sigma^{2}}e^{-\frac{x^{2}+\;y^{2}}{2\sigma^{2}}}$$

where *x* is the distance from the origin along the horizontal axis, *y* is the distance from the origin along the vertical axis, and *σ* is the standard deviation of the Gaussian distribution. Note that this standard Gaussian blur function is defined using the Euclidean distance, while the truncated Gaussian function (6) is defined differently using the minimal path distance along the contour from an input location *s*_0_. When applied in two dimensions, this formula produces a surface whose contours are concentric circles with a Gaussian distribution from the center point. After several experiments, we found that *σ* = 25 generated the most desirable smoothing effect for our DU contours.

After calculating G(x,z),thisvaluewas used to build a convolution matrix. When we applied this matrix to the original slice, it acted like a blurring filter; the original voxel's value receives the larger Gaussian value or weight while the neighboring pixels have smaller weights as their distance from the original voxel increases. After smoothing the slices in the axial plane (*x, y*), this formulation is applied on the *x* and *z* axis so that the contour is smoothed out in the sagittal plane also. Hence, combining the two Gaussian matrices along the axial plane and sagittal plane results in a more desirable result compared to the 3D Gaussian kernel approach.

As shown in Figure 2, the generated contours without any smoothing look coarse and noisy on the edge along the sagittal view (z‐direction). This happens due to the fact that the contours are generated slice‐by‐slice. When we apply the 3D Gaussian smoothing kernel, the edge in all views (directions) is over‐smoothed. This means that the contours in (b) don't retain the unique variations in (a) since the edge of the contours are overinflated and the generated contours are always larger than the clinician‐draw contour. Comparing the 3D filter in (b) with our method, we can see that the generated contours from our method are more desirable. Our contours in (c) are not overly smoothed and they still retain the unique variations that we saw in the noisy contours of (a). Hence, these contours in (c) result in distinct shapes which are not too similar to the red contours as if they were drawn by an actual human clinician with a different contouring style.

**FIGURE 3:**
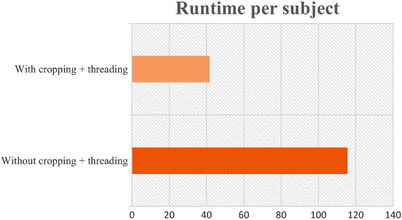
Runtime comparison of the PDUC model with and without cropping and multithreading. Without the cropping and threading method, the average runtime for each subject is 115.57 s (1.36 s per slice). With the cropping and threading method, the runtime is reduced to 41.64 s (0.50 s per slice).

**FIGURE 4:**
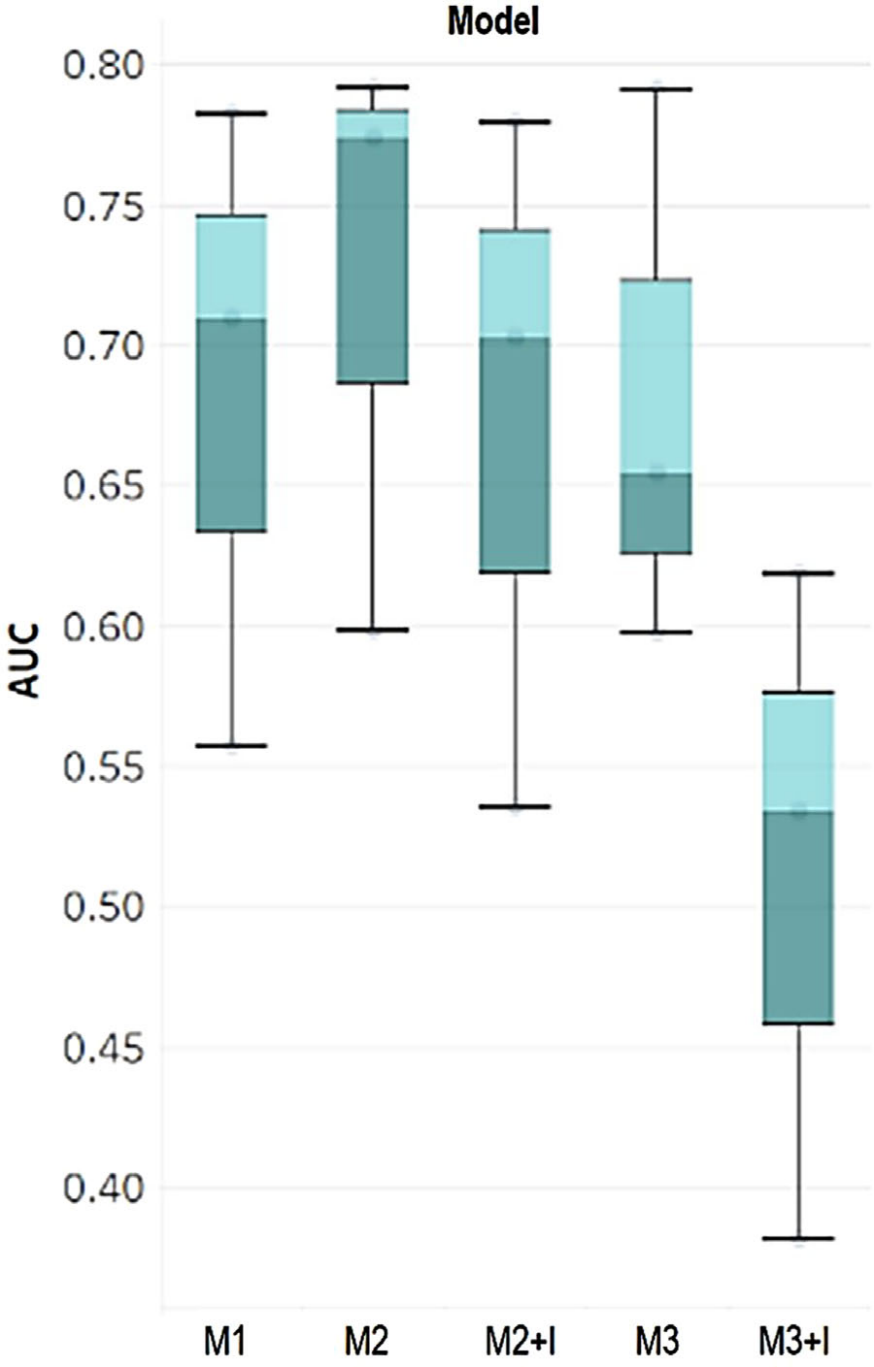
Boxplot of the AUCs from different models (defined in Table 2). M2 outperformed the other models in terms of classifying the binary editing level.

C. Auto‐filtering Layer:

Due to randomness, a fraction of the auto‐generated contours can deviate from the desired level of quality (realistic and clinically acceptable). For a practical use example, one may want to filter the generated DU contours and keep those in good quality. Thus, there is a need to build an auto‐filtering layer that helps filter out unacceptable contours and select only acceptable contours. Alternatively, clinical experts may visually inspect the generated DU contours one by one and filter out those unacceptable ones. However, this manual review process is time‐consuming and not realistic for large‐scale deployment.

After running the PDUC model on 14 subjects to generate the DU contours, we loaded the initial results into RayStation^®^ (by RayResearch Laboratories), where they were manually inspected by two radiation oncology residents in two separate blind review sessions. The two reviewers scored each contour on a scale of 1−5 using the clinical usability scale (CSC) and realism scale (RSC). CSC ranks how likely a contour can be used in the clinical setting, whereas RSC ranks how realistic a contour looks compared to a human‐drawn contour. These two scores are then classified into three levels of editing based on the criteria in Table 1. The editing level is calculated individually for each reviewer and jointly by taking the average scores from both reviewers.

**TABLE 1:** Evaluation metrics

CSC	RSC	Editing level
5	5	0 (no edits required)
3–4	3–4	1 (minor edits required)
1–2	1–2	2 (major edits required)

Clinical Usable Scores (USC) and Realistic Scores (RSC) are collected from the reviewers. These two scores are used to define the criteria for classifying the quality of the generated contours into three levels of required editing.

Due to randomness in our algorithm, there is a chance (varying with C) of generating clinically unacceptable contours. With the assumption that the clinically acceptable contours should be in a certain range of surface DSC and Hausdorff distance comparing with ground truth, we further explored how we could use the distance‐based metrics build an auto‐filtering model. The distance‐based metrics used in this part of the evaluation were 95% Hausdorff distance (HD) and surface DSC, with a tolerance of 3 mm. In addition, we also consider the adjustable variation parameter C. After selecting the potential covariates, we use logistic regression for our modeling.

For our auto‐filtering layer, we built several models from different combinations of the available covariates. M1 is a model that includes the C parameter only. M2 includes both C and surface DSC. M3 includes all covariates of C, surface DSC, and Hausdorff distance. We used *“+I”* to mark the model with interaction terms. To select the best classification model, we used the evaluation metric of AUC. With a limited sample size of 14 subjects, we split the data into a training‐testing ratio of 80:20. In the training set, we fitted multiple models with different combinations of the three covariates as well as their interaction terms. We eventually ran these models on the testing set and collected the AUC score for comparison. These results are summarized in Table 2.

**TABLE 2:** AUC of logistic regression model

	M1	M2	M2+I	M3	M3+I
Bladder	0.557	0.598	0.535	0.597	0.618
Prostate	0.782	0.774	0.779	0.654	0.381
Rectum	0.709	0.792	0.702	0.791	0.534

M1 is a model that includes the C parameter only.

M2 includes both C and surface DSC.

M3 includes all covariates of C, surface DSC, and Hausdorff distance.

We used “+I” to mark the model with interaction terms.

As shown in Table 2 and Figure 3, 4, model M2 with C and surface DSC outperformed the other models in terms of classifying the binary editing level. Thus, we can use this model to automatically filter the major editing (or not clinically acceptable) contours.

From the boxplots, in Figure 4 we have insight into the performance of this best filtering model, M2, across all ROI and for each ROI. Looking at the AUC (0.724 ± 0.109), we can see that this filtering model has good generalization power since it performs well across all 3 ROI.

## 3 RESULTS

Testing the runtime on the Seadragon cluster, the PDUC algorithm initially takes 115.57 s for each subject (1.36 s per slice). To speed up the runtime, we decided to implement some cropping on target regions and multithreading to process multiple slices at once. With the cropping and threading method, the runtime is reduced to 41.64 s (0.50 s per slice). Hence, the accelerated algorithm runs 3 times faster than the initial one (shown in Figure 3).

**FIGURE 5:**
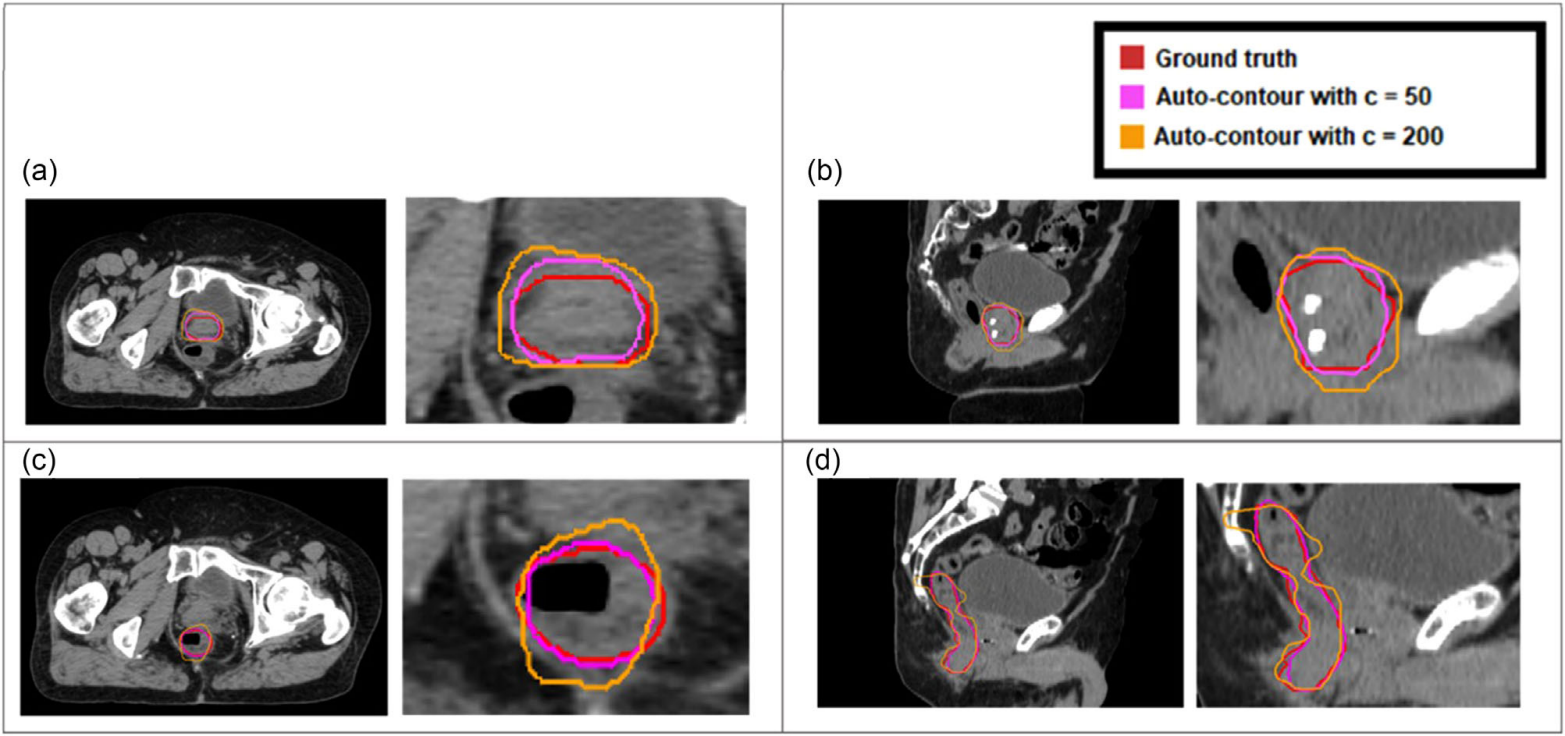
Example of auto‐generated DU contours of the prostate. The red line represents the clinician‐drawn contour, the pink line represents the auto‐generated DU contour with variation parameter c of 50, and the orange line represents the auto‐generated DU contour with c of 200. Plotting the three contours on top of one another, we can easily see how the DU contours are a variation of the ground truth contour and the effect of parameter c on the variation levels of the DU contour. (a) A slice from the axial view from subject 1; (b) A slice from the sagittal view from subject 1; (c) A slice from the axial view from subject 10; (d) A slice from the sagittal view from subject 10.

We evaluated the PDUC model for auto‐contouring of three ROI (prostate, bladder, and rectum) in 14 different patients. There are five different variation C levels (mentioned in formula (3)) for each ROI, which are 5, 50, 100, 200, and 300. Examples of auto‐generated DU contours using C values of 50 and 200 are shown in Figure 5. In the clinical review process, two radiation oncology residents provided feedback in independent blind review sessions. In these sessions, the C values of each DU contour were hidden, and the order of contours was randomly shuffled. Besides the auto‐generated DU contours, these reviewers also had access to the clinician‐drawn (ground‐truth) contours for reference. The filtering model has good generalization power since it performed well across all three ROI. Hence, integrating this filtering model as an additional layer of the PDUC model can reduce the time and labor needed for human manual inspection.

To calculate the percent agreement between the two reviewers, we compared their submitted editing level scores as defined in Table 1. Using Kappa statistics, we had a percent agreement of 0.71±0.03 across three ROI.^23^

**FIGURE 6:**
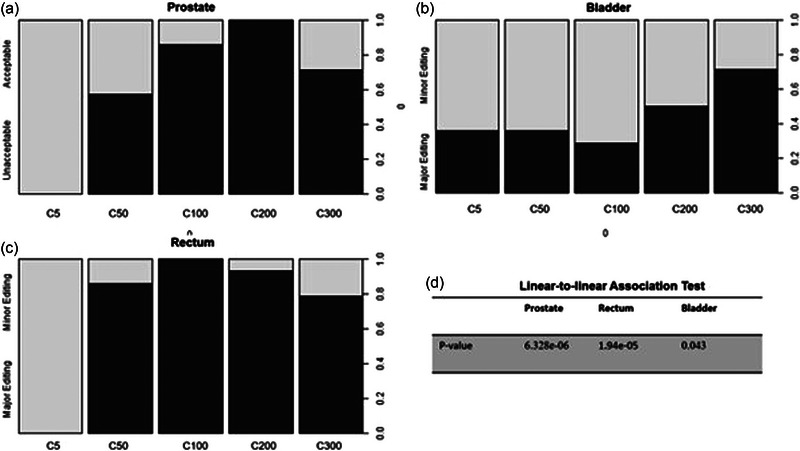
Spine plots comparing the proportions of minor/major editing levels (as defined in Table 1) across different variation parameters C. Separate plots were generated for (a) prostate; (b) bladder; (c) rectum. (d) The *p*‐values for testing associations between C and edit levels were done by linear‐to‐linear association test (sometimes known as ordinal Chi‐square test).

To evaluate the performance of parameter C, we calculated the editing proportion, which is the number of subjects in each editing level divided by the total number of subjects. Since no subject meet the “no editing” criteria as described in Table 1, we only focused on the minor/major editing level for our analysis. Then, we need to check for the relationship between the minor/major editing levels and the parameters C Since these two variables are both ordered categorical data, the linear‐to‐linear test, which is an extension of the Chisquare test for ordinal data, is appropriate here. The *p*‐value of each ROI of the prostate, rectum, and bladder in Figure 6d. From this figure, the parameter C indeed changes the proportion of minor/major editing significantly since the tested *p*‐values are ⩽0.05 in all three ROI. From Figure 6a, the parameter of 5 and 50 tends to produce minor‐editing (acceptable) contours for the ROI of the prostate. In Figure 6b, we see that all parameters C perform well for the bladder, except for C300. Hence, the PDUC model tends to produce acceptable contouring results for this particular ROI. In contrast, in Figure 6c, the model seems to struggle with the rectum since the proportion of major‐editing (unacceptable) contours are high in most parameters C, except for C5. Overall, C values of 5 and 50 consistently produced high proportions of contours needing only minor editing across all ROI compared to the other parameters C (proportions of 0.936 ± 0.111 and 0.552 ± 0.228 for C values of 5 and 50, respectively).

## 4 DISCUSSION

Using local contrast information from CT scans and clinician‐drawn contours, we created a parametric‐based model that can automatically generate the alternative structures of ROI in the lower abdominal region. With this automatic tool, we can generate a large dataset of DU contours that are not easily obtained using the manual process. This dataset can serve as the basis for future studies of treatment planning and quality assurance.

In this first paper, we manage to perform the manual review on 14 subjects. Since we have a good agreement of 0.75 in Kappa statistics between the two reviewers, we can see that 14 subjects are a good sample size for our quantitative analysis. From our parameter experimentation, our PDUC model performed best on the bladder, where we found the highest proportion of minor contour editing (0.606) compared with the prostate (0.364) and rectum (0.325). This might be due to the fact that the bladder has a more regular structure than the prostate and rectum. With a more complex structure like the prostate, the model is inconsistent at producing acceptable contours with minor editing for clinical use. Hence, more fine‐tuning of the model parameters is needed so that the model can account for the complex structures of the rectum and prostate, which vary more significantly in each testing subject. Because of this limitation, we also explored building an auto‐filtering model for major‐editing or “clinically unacceptable” DU contours. We also find that the C parameter and surface DSC together have the greatest impact on the editing level. In addition, integrating the filtering model as an additional layer of the PDUC model can reduce the time and labor for human manual inspection.

One big application of our model is in the space of quality assurance in treatment planning. As we mentioned earlier, the process of manually contouring for annotation was highly labor‐intensive and time‐consuming. This tool can aid in the process of auto‐generation of a large dataset of DU contours with clinically acceptable quality from our existing patient database. Depending on our parameter adjustment, we can generate the acceptable by picking parameters C of 5 and 50, or unacceptable DU contours with parameter C above 100. The automatic dataset saves time and eliminates the human bias in DU contours introduced by clinicians. From this large dataset, several group members can work on developing tools to automatically flag incorrect contouring or minimize these errors. With a larger autogenerated dataset, we expect to outperform existing methods^8^, ^9^, ^10^, ^11^, ^12^, ^13^, ^14^, ^15^, ^16^, ^17^ which use manual‐drawn contours. Additionally, our tool may be useful for robust treatment planning,^24^, ^25^, ^26^ by assessing the impact of various auto‐generated contours that represent geometric uncertainties in the contouring processes.

We present these results and applications as proof of concept rather than as a demonstration of efficacy. Although this model is built on the lower abdominal dataset, this parametric model is further expanded to other organs with some changes in the parameter tuning. Several methods are also built on the main design of fixed parameters and generalize to other modalities through hierarchical statistical atlases, or a set of interdependent rules and empirical decisions.^27^, ^28^As we develop this parametric model into a complete software tool, different generalization rules for parameter selection will be carefully discussed in our manual documentation.

## AUTHOR CONTRIBUTIONS

Vi Ly contributed to study design, data collection, analysis, and interpretation, and drafted the manuscript. Lizhong Liu contributed to model design. Daniel El Basha and contributed to data collection. Sean Maroongroge and Brian De contributed to clinical review of the results and revision of the manuscript. Carlos Cardenas, Laurence Court, and Xi Luo contributed to study design, study supervision and data interpretation, and critical revision of the manuscript. All authors discussed the results and gave final approval of the submitted article.

## CONFLICT OF INTEREST STATEMENT

Fundings from Varian Medical Systems.
